# A Predictive Disease Risk Model for Ankylosing Spondylitis: Based on Integrated Bioinformatic Analysis and Identification of Potential Biomarkers Most Related to Immunity

**DOI:** 10.1155/2023/3220235

**Published:** 2023-04-27

**Authors:** Wenxin Gao, Ruirui Hou, Yungang Chen, Xiaoying Wang, Guoyan Liu, Wanli Hu, Kang Yao, Yanke Hao

**Affiliations:** ^1^Shandong University of Traditional Chinese Medicine, Jinan, Shandong Province, China; ^2^Jinan Vocational College of Nursing, Jinan, Shandong Province, China; ^3^Affiliated Hospital of Shandong University of Traditional Chinese Medicine, Jinan, Shandong Province, China; ^4^The Second Affiliated Hospital of Shandong University of Traditional Chinese Medicine, Jinan, Shandong Province, China

## Abstract

**Background:**

The pathogenesis of ankylosing spondylitis (AS) is still not clear, and immune-related genes have not been systematically explored in AS. The purpose of this paper was to identify the potential early biomarkers most related to immunity in AS and develop a predictive disease risk model with bioinformatic methods and the Gene Expression Omnibus database (GEO) to improve diagnostic and therapeutic efficiency.

**Methods:**

To identify differentially expressed genes and create a gene coexpression network between AS and healthy samples, we downloaded the AS-related datasets GSE25101 and GSE73754 from the GEO database and employed weighted gene coexpression network analysis (WGCNA). We used the GSVA, GSEABase, limma, ggpubr, and reshape2 packages to score immune data and investigated the links between immune cells and immunological functions by using single-sample gene set enrichment analysis (ssGSEA). The value of the core gene set and constructed model for early AS diagnosis was investigated by using receiver operating characteristic (ROC) curve analysis.

**Results:**

Biological function and immune score analyses identified central genes related to immunity, key immune cells, key related pathways, gene modules, and the coexpression network in AS. Granulysin (GNLY), Granulysin (GZMK), CX3CR1, IL2RB, dysferlin (DYSF), and S100A12 may participate in AS development through NK cells, CD8^+^ T cells, Th1 cells, and other immune cells and represent potential biomarkers for the early diagnosis of AS occurrence and progression. Furthermore, the T cell coinhibitory pathway may be involved in AS pathogenesis.

**Conclusion:**

The AS disease risk model constructed based on immune-related genes can guide clinical diagnosis and treatment and may help in the development of personalized immunotherapy.

## 1. Introduction

Ankylosing spondylitis (AS) is an immune-mediated chronic inflammatory arthropathy. Most of its symptoms include damage to the axial bones, sacroiliac joints, and spinal attachment points, resulting in loss of joint function and even disability [1]. AS can easily cause inflammatory back pain and spread to the spine and sacroiliac joints, which significantly influences patients' quality of life, but early diagnosis and therapy can help to delay disease progression [2]. At present, the main drug treatments for AS include nonsteroidal anti-inflammatory drugs (NSAIDs), traditional synthetic antirheumatic drugs (DMARDs), and biological agents. NSAIDs are the first-line drugs for the treatment of AS. However, with the rapid development of bioengineering and genetic engineering, breakthroughs have been made in research on biological products. Biological treatments, such as tumour necrosis factor-*α* (TNF-*α*) inhibitors (TNFis) and IL-17A blockers, are commonly utilized in the clinic and have been shown to be effective [3]. TNF inhibitors have been demonstrated to reduce the concentrations of ESR and serum C-reactive protein in patients with active AS in some trials. Injecting them in the early phases of illness development may be more beneficial than later injections [4]. However, the pathogenesis and physiological and pathological mechanisms of AS have not been thoroughly studied, and there is a lack of accurate and detailed indicators for the early diagnosis of this disease. Therefore, exploring potential biomarkers for the early diagnosis of AS and developing a predictive risk model for this disease provides a new strategy for the early diagnosis and effective prevention and treatment of AS.

High-throughput sequencing and microarray analyses of human disease samples have generated a considerable amount of bioinformatic data in recent years, which has been useful in understanding the molecular mechanisms involved in related biological processes [5]. Furthermore, high-throughput microarray technology is frequently employed to explore and uncover promising biomarkers for disease diagnosis and prognosis at the genomic level [6]. Many studies have revealed that the innate immune system plays key roles in the onset and progression of AS and that it activates a wide range of immunological pathways. In addition, innate and innate-like immune cells, including *γδ* T cells, group 3 innate lymphoid cells (ILC3s), and neutrophils, show abnormal activity [7]. IL-17A and IL-17A-producing T cells are important mediators of various autoimmune disorders [8]. According to published studies, the quantities of IL-17A and T helper 17 (Th17) cells in the blood and synovial fluid of AS patients are much higher than those in healthy individuals, while CD8^+^ T cells are found in the synovial fluid of the inflamed joints of AS patients, and their levels are associated with disease activity [9]. Therefore, it is necessary to conduct a more comprehensive analysis of the molecular mechanism and potential immune microenvironment to deepen the understanding of AS.

The goals of this project were to identify promising biomarkers for the early diagnosis of AS and to develop a predictive disease risk model to improve diagnostic and treatment efficiency. This study analysed the gene expression profiles of patients with AS in the public Gene Expression Omnibus (GEO) database and conducted acomprehensive bioinformatic analysis to identify early molecular changes and biological mechanisms. Single-sample gene set enrichment analysis (ssGSEA) was used to assess immune cells and immunological functions. The genes that were found to be strongly expressed were screened, and their relationships with immune cells and immunological functions were investigated. We identified the prospective biomarkers most relevant to immunity in AS by analysing the area under the receiver operating characteristic (ROC) curve (AUC).

## 2. Materials and Methods

### 2.1. Data Acquisition and mRNA Filtering

The AS-related datasets GSE25101 and GSE73754 were selected from the GEO database (https://www.ncbi.nlm.nih.gov/geo). These two datasets were obtained by using whole genome microarray technology, including total RNA, and both include whole-blood samples of the peripheral blood. GSE25101 was generated using the GPL6947 platform Illumina HumanHT-12 V3.0, and GSE73754 was generated using the GPL10558 platform Illumina HumanHT-12 V4.0 expression beadchip. The GSE25101 dataset consisted of 32 samples, representing 16 AS patients with active disease and 16 sex- and age-matched controls, while the GSE25101 dataset contained 72 samples, representing 52 AS patients with active disease and 20 healthy controls. Since the two datasets were full RNA datasets, we used an algorithm to screen mRNA genes for follow-up analysis.

### 2.2. Difference Analysis

We extracted the relevant data from the gene matrix for the original mRNA expression profile dataset. For differential expression analysis of the sample dataset, the R program limma was employed, and the screening criteria were set to an adjusted *P* value (adj.*P*. val) < 0.05 and |log2 − fold change (FC)| > 1.

### 2.3. Coexpression Analysis

We merged and preprocessed the two datasets with the program named affy in R software, including performing background calibration, normalization, and log2 transformation. The “WGCNA” package was used to analyse the weighted coexpressed genes in the merged and corrected data.

### 2.4. Immune Scores

Coexpressed genes were scored by the ssGSEA algorithm, and immune cells and immunological functions were also scored separately. We used ssGSEA, which used an enrichment score to indicate the absolute degree of gene set enrichment in each sample due to a certain dataset [10]. This can be used to calculate a standardized enrichment score for each immune category. We used the GSVA [11], GSEABase, limma, GGPUBr, and reshape2 packages to conduct immune scoring of immune data. R software was used to draw a heatmap to show the correlations of samples with immune cells and immunological functions.

### 2.5. Correlation Analysis of Immune Cells and Immunological Functions

We assessed the relationships between immune cells and immunological functions by using the immune scores obtained as described above.

### 2.6. Difference Analysis of Immune Scores

The difference in immune scores between the normal group and the disease group was analysed with R software.

### 2.7. Analysis of the Correlations of Significantly Expressed Genes with Immune Cells and Immunological Functions

We identified significantly expressed genes by difference analysis of genes in the combined dataset and selected the log FC values of the two datasets for difference analysis to select several genes with higher expression scores. The R software psych package was used for analysis. The relationships between important genes and immune cells or immunological functions were investigated. Several genes most related to immunity were screened for follow-up analysis.

### 2.8. Construction of a Gene Model

From the previous step, the most relevant genes were selected for the construction of a gene model according to the degree of immune correlation. The Rms and ROCR packages of R software were used for analysis. First, the weighted coexpression data of the most related genes were screened, and then they were combined with clinical grouping data to determine the correlation scale of points with a high expression value and low expression value for each gene. The AUC index was calculated with the model, and an AUC regression chart was drawn.

## 3. Results

### 3.1. Differential Genes

**Figure 1:**
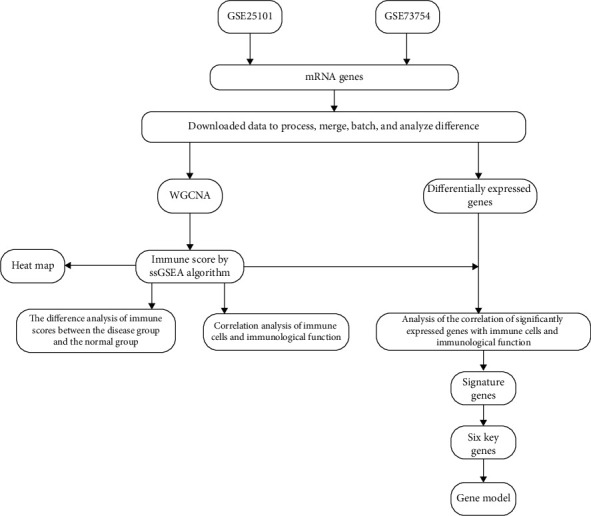
Flow chart of the study.

**Figure 2:**
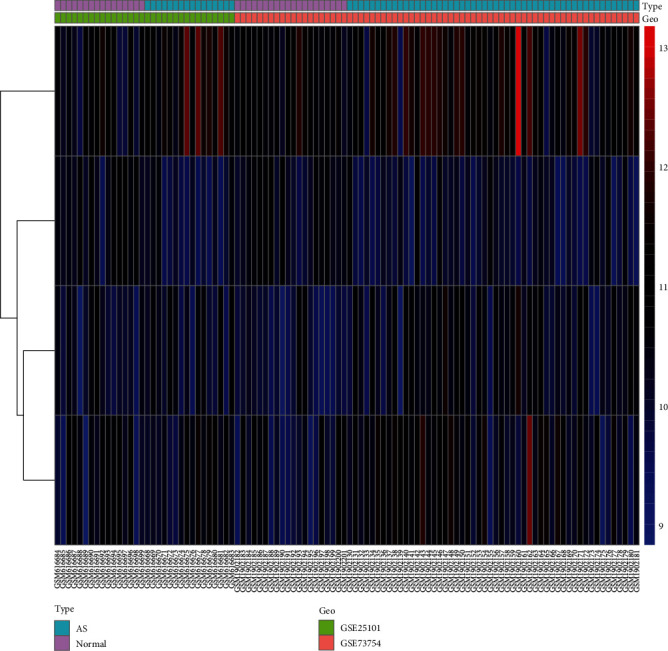
Differentially expressed gene (DEG) analysis of the integrated dataset. Heatmaps of control samples in the AS and normal groups in the GSE25101 and GSE73754 datasets.

Based on difference analysis of merged and corrected data, 4 differentially expressed genes (DEGs) IL2RB, dysferlin (DYSF), S100A12, and NRGN were identified. The expression of IL2RB was downregulated, while that of DYSF and S100A12 was upregulated (Flow chart of the study was shown in Figure 1). According to the difference analysis of the two datasets studied, there were 36 and 163 differentially expressed genes in GSE25101 and GSE73754, respectively, and 199 genes when combined (Figure 2).

### 3.2. Gene Screening

A total of 9595 weighted coexpressed genes were screened from the two datasets by weighted gene coexpression network analysis (WGCNA).

### 3.3. Immune Scores

**Figure 3:**
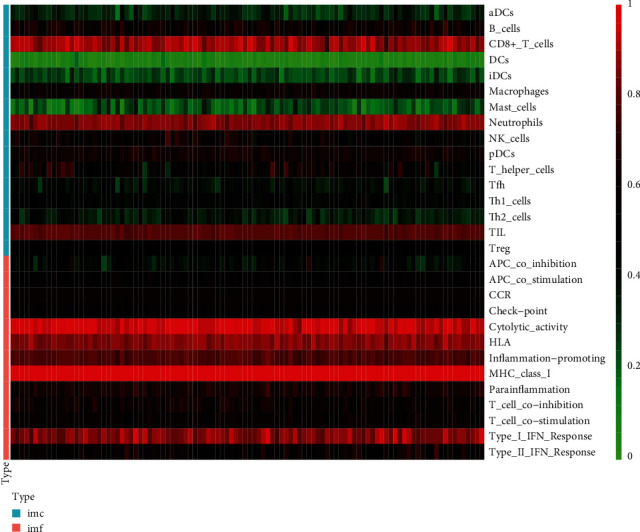
After calculation, the score of the combined sample in the immunoassay was obtained, and the immune score result was obtained. A heatmap was drawn according to the immune score. The darker the red colour is, the higher the immune score was, and the higher the level in the sample was. In contrast, the darker the green colour is, the lower the immune score was, and the lower the level in the sample was.

After calculation, the score of the combined sample in the immunoassay was obtained, and the immune score result was obtained. A heatmap was drawn according to the immune score. We found that the levels of CD8^+^ T cells, neutrophils, tumour-infiltrating lymphocytes (TILs), cytokine activity, HLA, inflammation promotion, MHC class I, and the type I IFN response were high in our samples (Figure 3).

### 3.4. Correlation Analysis of Immune Cells and Immunological Functions

**Figure 4:**
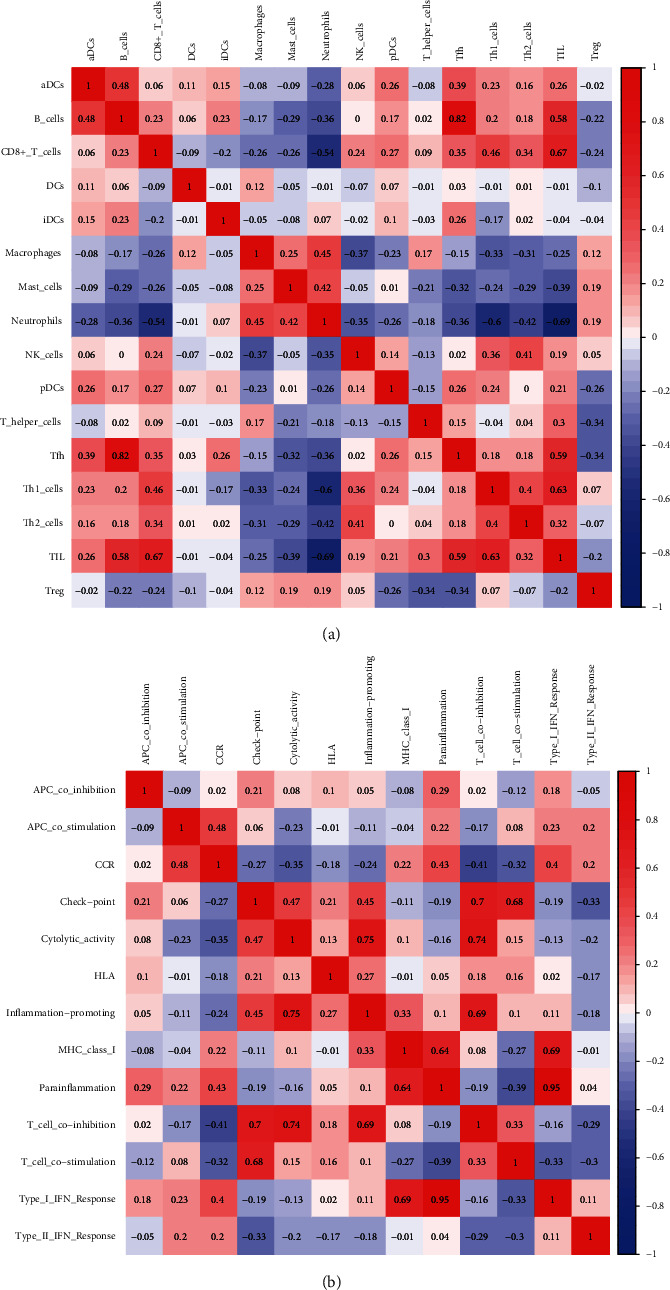
Correlation analysis of immune cells and immunological functions. The darker the red colour is, the higher the value was, and the stronger the positive correlation was. In contrast, the darker the blue colour is, the higher the value was, and the stronger the negative correlation was. (a) Correlation analysis of immune cells. (b) Correlation analysis of immunological functions.

By correlation analysis, we found that among immune cells, there were positive correlations between B cells and T follicular helper (Tfh) cells (*r* = 0.82) and between CD8^+^ T cells and TILs (*r* = 0.67) and negative correlations between neutrophils and TILs (*r* = 0.69) and between neutrophils and T helper 1 (Th1) cells (*r* = 0.6) (Figure 4(a)). Correlation analysis of immunological functions showed that there were positive correlations between the type I IFN response and parainflammation (*r* = 0.95), between inflammation promotion and cytolytic activity (*r* = 0.75), between T cell coinhibition and cytolytic activity (*r* = 0.74), and between T cell coinhibition and checkpoint (*r* = 0.7). There were also negative correlations between inflammation promotion and CCR (*r* = 0.41) and between T cell costimulation and parainflammation (*r* = 0.39) (Figure 4(b)).

### 3.5. Difference Analysis of Immune Scores

**Figure 5:**
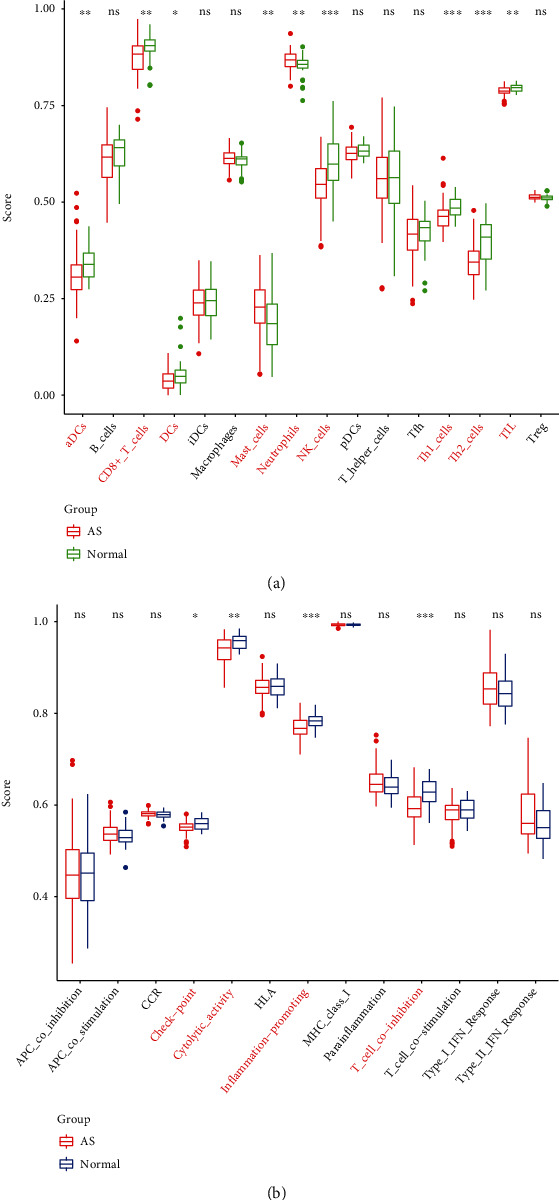
Difference analysis of immune scores between the normal and AS groups. (a) Difference analysis of immune cell scores between the normal and AS groups. Red indicates the disease group, and green indicates the normal group. Asterisks are used to represent *P* values that indicate that the observed difference was significant (the symbol ^∗^ represents a *P* value < 0.05, the symbol ^∗∗^ represents a *P* value < 0.01, and the symbol ^∗∗∗^ represents a *P* value < 0.001). (b) Difference analysis of immunological function scores between the normal and AS groups. Red indicates the disease group, and blue indicates the normal group. Asterisks are used to represent *P* values that indicate that the observed difference was significant (the symbol ^∗^ represents a *P* value < 0.05, the symbol ^∗∗^ represents a *P* value < 0.01, and the symbol ^∗∗∗^ represents a *P* value < 0.001).

In the difference analysis of immune cell scores, there were significant differences in activated dendritic cells (aDCs), CD8^+^ T cells, dendritic cells (DCs), mast cells, neutrophils, natural killer (NK) cells, Th1 cells, T helper 2 (Th2) cells, and TILs between the disease group and the normal group (Figure 5(a)). In the score difference analysis of immune functions, there were significant differences in the checkpoint, cytolytic activity, inflammation promotion, and T cell coinhibition between the disease group and the normal group (Figure 5(b)).

### 3.6. Analysis of the Correlations of Significantly Expressed Genes with Immune Cells and Immunological Functions

**Figure 6:**
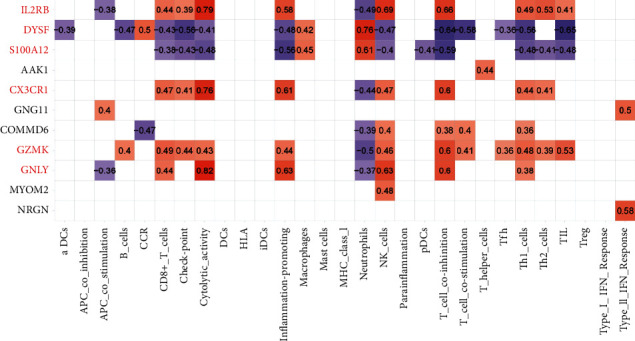
Analysis of the correlations of significantly expressed genes with immune cells and immunological functions. The darker the red colour is, the higher the value and the stronger the positive correlation were, and vice versa, the lighter the red colour is, the weaker the positive correlation was. In contrast, the darker the purple colour is, the higher the value and the stronger the negative correlation were, and vice versa, the lighter the purple colour is, the weaker the negative correlation was. NK cells and T cell coinhibition were strongly correlated with the significantly expressed genes. CD8^+^ T cells and Th1 cells were strongly correlated with the significantly expressed genes.

According to the screening criteria, we screened the following DEGs: neurogranin (NRGN), MYOM2, granulysin (GNLY), granzyme K (GZMK), COMMD6, GNG11, CX3CR1, AAK1, IL2RB, dysferlin (DYSF), and S100A12. We found that there were strong correlations between Th1 cells, NK cells, or T cell coinhibition and the above genes (Figure 6).

### 3.7. Construction of the Gene Model

**Figure 7:**
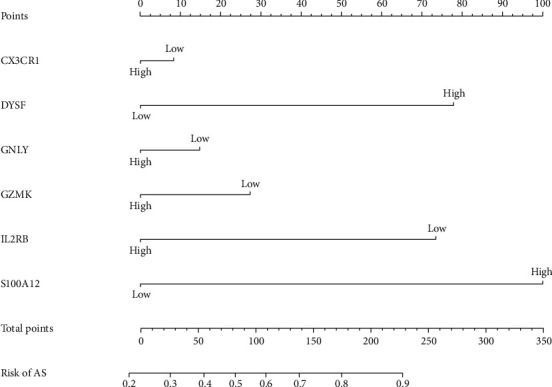
A gene prediction model based on immune analysis correlations was constructed. The nomogram was composed of GNLY, GZMK, CX3CR1, IL2RB, DYSF, and S100A12.

**Figure 8:**
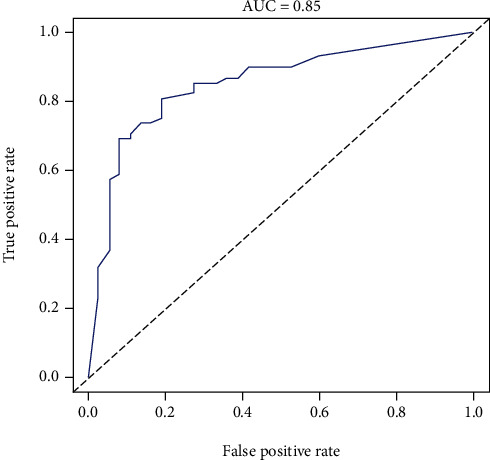
ROC curve analysis. According to the AUC results (AUC = 0.85), the predictive ability of the model was relatively accurate.

**Figure 9:**
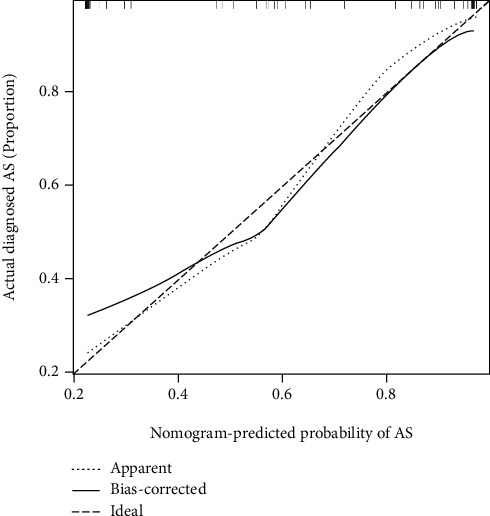
Use of the internal dataset to create a calibration chart to evaluate the accuracy of the model. The results showed that the model was accurate.

We selected the six genes with the highest correlations determined by the immunoassay, GNLY, GZMK, CX3CR1, IL2RB, DYSF, and S100A12, to construct a gene model. According to the weighted coexpression data of the most related genes and combined with clinical grouping information, we constructed a gene prediction model based on the immunoassay correlations (Figure 7). ROC curve analysis (AUC = 0.85) of the model proved its promising predictive value for AS (Figure 8). In addition, a calibration chart was drawn to evaluate the model (Figure 9).

## 4. Discussion

As a chronic and progressive form of arthritis, AS usually occurs in people before the age of 45, resulting in limited physical function, a significant decline in work efficiency, a serious negative impact on quality of life, and adverse psychological and physiological effects [12]. Previous bioinformatic studies have found that there are significant differences in CD8^+^ T cells, native CD4^+^ T cells, and other immune cells between AS patients and healthy controls and that these variations are strongly linked to the onset and progression of AS [13]. IL-17 is the only type 3 immune cytokine that has been successfully targeted in AS [14], and a large amount of clinical trial data has proven that secukinumab, a monoclonal antibody specific for IL-17, can have the same effect as TNF inhibitors in the treatment of AS patients [15]. These investigations have demonstrated that the immune system and immune cells play critical roles in the pathogenesis of AS, although additional molecular targets and potential mechanisms of immune-related AS remain unknown. We believe that immune-related genes may play critical roles in regulating the onset and progression of AS. As a result, more research in this field is needed.

In this study, by comparison with the healthy control group, we identified four DEGs from two datasets, and WGCNA was used for gene screening. We assessed coexpression genes, immune cells, and immunological functions using ssGSEA, which was utilized to demonstrate the associations of samples with immune cells and immunological functions. Then, on the basis of immune scores, we analysed the correlations between immune cells and immunological functions and used R software to analyse the correlations of significant genes with immune cells and immune functions. Finally, six key immune-related genes, GNLY, GZMK, CX3CR1, IL2RB, DYSF, and S100A12, were screened to construct a predictive gene model based on the immunoassay correlations. The sensitivity and specificity of the genes in the model for the diagnosis of AS were investigated using ROC curve analysis. In addition, calibration diagrams were used for internal verification. The findings revealed that the genes tested in this study have the potential to be employed as promising biomarkers for the early diagnosis and treatment of AS and that the level of gene expression can predict the occurrence of AS.

Through many screening processes, such as difference analysis, immune scoring, and correlation analysis, we selected GNLY, GZMK, CX3CR1, IL2RB, DYSF, and S100A12 as the key genes. ROC curve analysis and calibration map evaluation showed that these genes may be the most relevant early diagnostic markers in terms of immunity in AS. While IL2RB and S100A12 have been reported in the literature to be related to the pathogenesis of AS, the relationships of the other genes to AS have not been clearly reported. Therefore, this model represents a new discovery. GNLY is a member of the AMP cell lysate and proinflammatory peptide family. This peptide is found in the granules of T cells and NK cells, and it is generated along with granzyme and perforin by these cells [16]. GNLY acts as an endogenous danger signal under inflammatory conditions, recruiting and activating leukocytes, including antigen-presenting cells (APCs), through Toll-like receptor 4 (TLR4), to promote antigen-specific immune responses [17]. Some studies have shown that GNLY accelerates the deterioration of psoriatic arthritis and that its apoptosis-related mechanism mediates the development of joint lesions [18]. GZMK is a granule secretase belonging to the serine protease family. A large amount of experimental data has shown that GZMK is cytotoxic and may promote the release of proinflammatory cytokines [19]. Mogilenko et al. [20] found that GZMK-expressing CD8^+^ T cells existed in mice as a unique cell subset that developed under the influence of an ageing environment and promoted inflammatory factor expression by increasing the secretion of GZMK, which emphasized that GZMK^+^CD8^+^ T cells and GZMK could be potential targets in the treatment of age-related immune system disorders. In a recent bioinformatic study, GZMK was identified as a potential marker for the early diagnosis of rheumatoid arthritis (RA), and the analysis showed that GZMK might trigger continuous inflammatory expansion in RA [21]. CX3CR1 is a chemokine receptor expressed on monocytes and a key regulator of monocyte adhesion and migration [22]. The M2 phenotype identifies an alternatively activated subset of monocytes/macrophages, and M2-like monocytes can be defined as CX3CR1^+^CD163^+^/CD206^+^ cells [23, 24]. Zhao et al. [25] found that the M2 phenotype was the dominant phenotype of monocytes/macrophages in the local tissues and peripheral blood of patients with advanced AS and further confirmed that the pathology of advanced AS was characterized by tissue repair and tissue remodelling. Some studies have shown that in patients with AS, CX3CR1^+^ monocytes have a specific proinflammatory transcriptome and actively participate in the activation and expansion of ILC3s, thus promoting the persistent proinflammatory status of AS [26]. The IL2RB gene is a cytokine signalling-related gene [27] that encodes the receptor of interleukin 2 (IL-2) and is associated with autoimmune and inflammatory diseases [28]. A study found that an IL2RB polymorphism (rs2281089) significantly reduced the risk of RA [28]. A 10-year follow-up study of AS patients found that 14 single-nucleotide polymorphisms (SNPs) in 10 different genes distributed in AS patients were significantly associated with peripheral arthritis (PA) and that IL2RB was one of the 10 genes [29]. DYSF is a type II transmembrane glycoprotein in the ferlin family. The dysregulation of DYSF expression is closely related to many hereditary myopathies and autoimmune diseases [30, 31]. Xiao et al. reported that DYSF is crucial in the disease progression of dermatomyositis (DM) and polymyositis (PM), two subgroups of idiopathic inflammatory myopathy (IIM). Upregulated expression of DYSF, together with HLA-An and MCP-1, was found to play crucial roles in inflammatory cell infiltration and muscle injury [31]. S100A12, a member of the S100 protein family, has unique proinflammatory activity and is significantly expressed in a variety of inflammatory myopathies [32]. Recently, S100A12 was reported to be significantly expressed in the plasma of patients with Blau syndrome (BS, a dominant hereditary autoinflammatory disease), the number of active joints was strongly linked with the amount of S100A12, and S100A12 was discovered to be a biomarker of joint disease activity in BS patients [33]. A multicentre prospective cohort study performed in France found that changes in the S100A12 expression levels in patients with RA were a good predictor of the clinical therapeutic efficacy of TNFis in RA and created a multivariate model to accurately predict RA patient responses to TNFis that can be used in everyday practice for individualized treatment [34]. In summary, GNLY, GZMK, CX3CR1, IL2RB, DYSF, and S100A12, which we selected for inclusion in our model, are closely related to autoimmune and inflammatory diseases, especially in the regulation of joint inflammation.

For the early diagnosis and treatment of patients with AS, it is critical to monitor and restore immune system function. NK cells, a kind of innate immune cell, are large granular lymphocytes that differentiate from common lymphoid progenitors [35]. NK cells are cytotoxic and can release a vast range of cytokines that contribute to immune response modulation [35]. Increasing evidence shows that NK cells play important roles in the pathogenesis and disease development of AS. Kucuksezer et al. summarized a substantial number of studies [35], reporting that HLA-B27, ERAP-1, KIRS, and other AS-related genes can potentially affect the function of NK cells and that some changes in NK cell function can be used to anticipate the response to therapy in patients with AS. According to a study, TNF-*α* secretion by autologous monocytes was enhanced by circulating CD56bright NK cells in AS patients, and this could contribute to the persistent invasive immunological process in AS, which led to aberrant inflammation [36]. Jiao et al. studied the gene polymorphisms in NK cell receptors and found that due to genetic factors related to HLA-B27, the variation in KIRS and its corresponding specific HLA-C ligand might hinder the role of NK cells in recognizing and eliminating targets in the immune response, thus promoting the development of AS [37]. CD8^+^ T cells play a key role in the elimination of intracellular infections and malignant cells [38]. It can provide long-term protective immunity and is closely related to the pathogenesis of immune system diseases such as rheumatoid arthritis [38, 39]. Hanson et al. found that after stimulation of peripheral blood mononuclear cells of AS patients in vitro, the receptor characteristics of CD8^+^ T cells have changed, and the specific clones of EBV and CMV have significantly enlarged. This dynamic change of peripheral T cell response in AS patients suggests that adaptive immune disorder may be one of the pathological features of the disease [40]. Gracey et al. found that the decrease in the sheer number of CD8^+^ T cells in AS patients led to the loss of cytotoxic cell gene expression, and it was also found that CD8^+^ T cells preferentially gathered in the inflammatory joints of AS patients [41]. Cytolytic activity is an important process of cell development. Immunologic surveillance and cytolytic activity against transformed or infected cells may benefit from crosstalk between CD8^+^ T cells and NK cells, which can be recruited at different stages of immune control. NK cells and CD8^+^ T cells interact closely to induce specific cytolysis [42]. Th1 cells play a dominant role in helping the host defend against intracellular pathogens and are also involved in the development of some types of autoimmune diseases. Excessive stimulation of the Th1 T cell lineage may result in the production of a large number of proinflammatory cytokines, contributing to the chronic inflammatory state in AS [43]. Wang et al. found that the Th1/Th2 cell ratio was increased significantly in patients with AS and that this imbalance led to an increase in the mRNA and protein expression of immune mediators [44]. Wen et al. performed clinical experiments [45] and found that the levels of IL-4 and IL-10 in the serum of patients with active AS were significantly decreased, while the levels of TNF-*α* and IFN-*γ* were increased, suggesting that an imbalance in Th1/Th2 cells may be involved in the pathogenesis of AS. The changes in the expression of Th1 and Th2 cytokines in the peripheral blood of AS patients can reflect the activity of this disease. In conclusion, alterations in the numbers and activities of NK cells and Th1 cells are important in the progression of AS. They can reflect disease progression and activity and may have predictive value for the treatment and prognosis of AS.

A complex network of checks and balances, including costimulatory and coinhibitory pathways that regulate T cell activation and function, regulates the immune response [46]. Costimulatory and coinhibitory receptors, as well as their ligands, shape and regulate the immune response in fundamental ways. Costimulation and coinhibition regulate T cell functions, and an imbalance between them might cause tolerance to break down, leading to autoimmune disorders [47, 48]. The immunoglobulin superfamily (Ig-SF) and TNF receptor superfamily (TNFR-SF) contain the majority of costimulatory and coinhibitory molecules. Both receptor families are essential for T cell regulation and govern T cells in a dynamic and sequential manner [49]. After synthesizing the results of many studies, Simons et al. concluded that coinhibition seems to be the key to balancing T cell activation, protecting tolerance, and inducing immune homeostasis. Together with the costimulatory pathway, the coinhibitory pathway is an important mediator of T cell failure during immune responses to infection or cancer [50]. Zhang and Vignali concluded that a defect in the coinhibitory pathway was also related to RA susceptibility and progression and proposed the possibility that an inherent defect in coinhibition would lead to the loss of systemic tolerance in RA [47]. In our study, we found that T cell coinhibition was closely related to the genes selected for inclusion in our model and was related to most immune functions.

## 5. Conclusion

To summarize the above conclusions, based on the high-throughput GEO dataset, we obtained the genes GNLY, GZMK, CX3CR1, IL2RB, DYSF, and S100A12 as the genes most related to immunity and identified them as potential biomarkers for the early diagnosis of AS with the help of integrated bioinformatic analysis. These genes might affect AS through immune cells, such as NK cells, CD8^+^ T cells, and Th1 cells, and we found that the T cell coinhibitory pathway might be a potential component of the pathogenesis of AS. Additionally, we constructed an AS disease risk model composed of immune-related genes for the first time and evaluated the potential of the expression of these genes to predict the risk of AS. We concluded that monitoring the expression of GNLY, GZMK, CX3CR1, IL2RB, DYSF, and S100A12 in vivo might have good potential for early diagnosis of AS.

We aimed to find biomarkers for AS, learn more about the roles of immune cells and immunological functions in AS, and develop an AS disease risk model based on immune-related genes for early detection. There were some limitations to our research. First, due to the small data sample size and data content limitations, we could not carry out external dataset verification. Furthermore, when we built the model, we could not refer to additional factors, such as age and sex, for a more detailed group discussion. Second, the exact mechanism of the immune response induced by the genes used to construct the model needs to be further studied. Finally, there is a lack of clinical data and experiments to verify the levels of gene expression and their effects on disease.

## Data Availability

The datasets presented in this study can be found in online repositories at https://www.ncbi.nlm.nih.gov/geo/query/acc.cgi?acc=GSE73754 and https://www.ncbi.nlm.nih.gov/geo/query/acc.cgi?acc=GSE25101.
